# CHD7 Deficiency in “*Looper*”, a New Mouse Model of CHARGE Syndrome, Results in Ossicle Malformation, Otosclerosis and Hearing Impairment

**DOI:** 10.1371/journal.pone.0097559

**Published:** 2014-05-19

**Authors:** Jacqueline M. Ogier, Marina R. Carpinelli, Benedicta D. Arhatari, R. C. Andrew Symons, Benjamin T. Kile, Rachel A. Burt

**Affiliations:** 1 Murdoch Childrens Research Institute, Parkville, Victoria, Australia; 2 The HEARing Cooperative Research Centre, Parkville, Victoria, Australia; 3 Walter and Eliza Hall Institute of Medical Research, Parkville, Victoria, Australia; 4 Department of Medical Biology, University of Melbourne, Parkville, Victoria, Australia; 5 Department of Genetics, University of Melbourne, Parkville, Victoria, Australia; 6 Department of Paediatrics, University of Melbourne, Parkville, Victoria, Australia; 7 ARC Centre of Excellence for Coherent X-ray Science, Department of Physics, La Trobe University, Bundoora, Victoria, Australia; 8 Department of Ophthalmology, Royal Melbourne Hospital, Parkville, Victoria, Australia; University of Iowa, United States of America

## Abstract

CHARGE syndrome is a rare human disorder caused by mutations in the gene encoding chromodomain helicase DNA binding protein 7 (CHD7). Characteristics of CHARGE are varied and include developmental ear and hearing anomalies. Here we report a novel mouse model of CHD7 dysfunction, termed *Looper*. The *Looper* strain harbours a nonsense mutation (c.5690C>A, p.S1897X) within the *Chd7* gene. *Looper* mice exhibit many of the clinical features of the human syndrome, consistent with previously reported CHARGE models, including growth retardation, facial asymmetry, vestibular defects, eye anomalies, hyperactivity, ossicle malformation, hearing loss and vestibular dysfunction. *Looper* mice display an otosclerosis-like fusion of the stapes footplate to the cochlear oval window and blepharoconjunctivitis but not coloboma. Looper mice are hyperactive and have vestibular dysfunction but do not display motor impairment.

## Introduction

Syndromic hearing loss accounts for approximately half of all inherited hearing impairment [1], [2]. Over 400 syndromes involving hearing loss have been characterised [3], with most having an underlying genetic cause [1]. In 60% of reported cases a single point mutation is responsible for the disease [4]. CHARGE is a syndromic hearing impairment that is predominantly caused by mutations in the chromodomain helicase DNA binding protein 7 (*CHD7*) gene. CHARGE is an acronym describing some of the main clinical features of the disease (ocular **C**oloboma, **H**eart defects, choanal **A**tresia, **R**etarded growth, **G**enital hypoplasia and **E**ar anomalies). Numerous additional features are observed in the syndrome, including hyperactive behaviour [5], nerve anomalies, cleft palate, facial asymmetry and limb deformity [6]. The extent and variability of CHARGE characteristics exemplify the importance of CHD7 in multiple developmental pathways. The incidence of CHARGE syndrome is 1 in 8,500 to 10,000 live births [7].

CHD7 regulates gene expression, particularly that of transcription factor genes [8], [9], through ATP-dependent nucleosome remodelling [10]. Whilst the timing and tissue-specific expression of CHD7 has been investigated [9], [11], [12] little is known about the precise genes and pathways that it regulates. CHD7 binds distally with over 10,000 enhancing regulators [13], co-binding with factors including PBAF [9] and Sox2 [8], [14], potentially affecting numerous pathways driving early development. This explains to some extent the range of CHARGE characteristics [14].

Several *Chd7* mouse mutations have been reported, including two targeted, three gene-trapped, eleven chemically induced and one spontaneous allele (Table 1). Mutants exhibit relatively similar characteristics including a range of CHARGE-like anomalies with varying penetrance, although some differences between strains have been noted, and this may be due to allelic variation or differences in the genetic backgrounds on which these alleles have arisen [11]. Study of the phenotype of mice carrying nonsense mutations of *Chd7* indicates that the C-terminal NLS, BRK and SANT protein domains are functionally important. Similarly, nonsense mutations of *CHD7* lead to a more severe clinical presentation in patients [15]. We conducted a detailed phenotypic characterisation of a new mouse model of CHARGE, *Looper*, which carries an ethylnitrosourea (ENU)-induced point mutation in *Chd7.* We identified several phenotypic anomalies including growth retardation, facial asymmetry, eye anomalies and hyperactivity. The mice were hearing-impaired due to fusions of the stapes tubercle to the temporal bone, and of the footplate to the cochlear oval window. *Looper* mice also displayed hypoplasia of the semi-circular canals and vestibular dysfunction, but motor coordination appeared unimpaired. All of these phenotypic abnormalities have been previously observed in other CHARGE mutants. The coloboma observed in human patients has not been reported in mouse models. Herein, we provide evidence that CHD7-deficiency in mice does not result in coloboma in any structures of the eye.

**Table 1:** Published mutant alleles of *Chd7.*

Allele	Background	Derivation	Mutation	Effect	Ref
*Coa1*	C57BL/6J	ENU	c.2155A>T	p.K719X	[35]
*Cycn*	BALB/c×C3H/HeN	ENU	c.4286T>A	p.L1429X	[11]
*Dz*	BALB/c×C3H/HeN	ENU	c.5536G>T	p.E1846X	[11]
*Edy*	BALB/c×C3H/HeN	ENU	c.307C>T	p.Q103X	[11]
*Flo*	C3HeB/FeJ	ENU	c.5635+2T>C (IVS27+2T>C)	p.S1864X	[11]
*Gt(RRR136)Byg*	129P2/OlaHsd×C57BL/6	gene trap	Insertion inintron 4	reporter fusion	[36]
*Gt(S20-7E1)Sor*	129S4/SvJae×C57BL/6J	gene trap	Insertion in exon 1	reporter fusion	[12]
*Gt(XK403)Byg*	129P2/OlaHsd×C57BL/6	gene trap	Insertion inintron 36	reporter fusion	[36]
*Lda*	BALB/c×C3H/HeN	ENU	c.3195T>A	p.Y1066X	[11]
*Looper*	BALB/c	ENU	c.5690C>A,	p.S1897X	
*Mt*	BALB/c×C3H/HeN	ENU	c.5021–2A>G (IVS22–2A>G)	p.V1688X	[11]
*Obt*	BALB/c×C3H/HeN	ENU	c.3945T>A	p.Y1315X	[11]
*Ome*	BALB/cByJ	Spontaneous	Deletion ofexons 2 & 3	No proteinproduced	[29]
*tm1.1Dmm*	129S6/SvEvTac×129S1/Sv1mJ	Gene targeting	Floxed exon 2	No effect	[37]
*tm1.2Dmm*	129S6/SvEvTac×129S1/Sv1mJ×Swiss Webster	Gene targeting	Deletion of exon 2	No proteinproduced	[37]
*Todo*	BALB/c×C3H/HeN	ENU	c.2208+2T>C (IVS3+2T>C)	p.H539X	[11]
*Vlk*	C3HeB/FeJ	ENU	c.4377T>A	p.Y1459X	[38]
*Whi*	C3HeB/FeJ	ENU	c.2918G>A	p.W973X	[11]

Ref reference, ENU ethylnitrosourea. Build: GRCm38.p1, Reference: CCDS38689.1.

## Materials and Methods

### Ethics Statement

The WEHI and MCRI animal ethics committees approved this work in project numbers 2011.016 and A726 respectively.

### Mice

Colonies of mice were maintained at the Walter and Eliza Hall Institute of Medical Research (WEHI) and the Murdoch Childrens Research Institute (MCRI). Animals were group-housed in individually ventilated micro-isolator cages (Airlaw, Smithfield, NSW, Australia) or (Tecniplast, Buguggiate, VA, Italy). All boxes were enriched with a plastic house/toy and animals had a 10–12 hour dark period. Mice had access to standard Barastoc mouse chow (Ridley AgriProducts, Melbourne, VIC, Australia) and sterilized water *ad libitum*.

### Mutagenesis Screen

Male BALB/c mice were injected intraperitoneally with 85 mg/kg ENU (Sigma-Aldrich, Castle Hill, NSW, Australia) weekly for 3 weeks as previously described [16], [17]. After 12 weeks mice were mated with untreated BALB/c females to produce first generation (G_1_) progeny. Animals were screened by acoustic startle response (ASR) testing at 8 weeks of age. Mice with an ASR below 200 mV in response to white noise bursts of 115 dB SPL were test-mated to determine heritability of the phenotype. Mutant strains were serially backcrossed to BALB/c for 9 generations before phenotypic analysis. Mice were genotyped for the *Chd7^Looper^* mutation using the Amplifluor SNPs HT genotyping system FAM-JOE (Merck Millipore, Kilsyth, VIC, Australia) and the primers GAAGGTGACCAAGTTCATGCTGCTCTACTGGCCGAACACGTC (forward wild-type), GAAGGTCGGAGTCAACGGATTGCTCTACTGGCCGAACACGTA (forward mutant) and CGCCGGTCCGTCTTCATTAG (reverse).

### Acoustic Startle Response

Acoustic startle response (ASR) was measured using an SR-LAB system (San Diego Instruments, San Diego, CA, USA). Testing was conducted in the light phase of the light cycle and the testing environment was illuminated. Mice were restrained in a Perspex chamber and acclimatized to background white noise of 70 dB SPL for 1 min. Trials were presented in pseudorandom order and separated by intervals of 3–8 sec. Mice underwent 6 trials each of 70, 85, 90, 95 and 100 dB SPL and 16 trials of 115 dB SPL. Each white noise pulse was presented for 40 msec. After deleting the largest and smallest values, the average startle amplitude for each stimulus was calculated and plotted using Prism v 6.0b software (GraphPad Software Inc, La Jolla, CA, USA).

### Auditory Brainstem Response

The auditory brainstem response (ABR) of mice was tested using an evoked potentials workstation (Tucker Davis Technologies, Alachua, FL, USA) as described previously [16], [18]. Briefly, mice were anaesthetized by intraperitoneal injection of 100 mg/kg ketamine and 20 mg/kg xylazine (BALB/c background) or 100 mg/kg ketamine, 10 mg/kg xylazine and 3 mg/kg acepromazine (mixed genetic background) and eyes moistened with lacrilube. In the latter case 2.5 mg/kg atipamezole hydrochloride was injected at the completion of testing to aid recovery. A free-field magnetic speaker (model FF1, Tucker Davis Technologies) was placed 10 cm from the left pinna. Computer-generated clicks (100 µsec duration, with a spectrum of 0–50 kHz) and 3 msec pure tone stimuli of 4, 8, 16 and 32 kHz were presented with maximum intensities of 100 dB SPL. ABRs were recorded differentially using subdermal needle electrodes (S06666-0, Rochester Electro-Medical, Inc., Lutz, FL, USA) positioned at the vertex of the skull (+ve), in the left cheek (−ve) and the hind left leg (ground). ABRs were averaged over 512 repetitions of each stimulus and traces were analysed to determine ABR threshold using BioSig software (Tucker Davis Technologies). The threshold was defined as the lowest intensity stimulus that reproducibly elicited an ABR.

### Mutation Identification

Exome sequencing of two N_5_ *Chd7* ^+*/*Looper^ male mice was performed at the Australian Genome Research Facility (AGRF) using the 100803_MM9_exome_rebal_2_EZ_HX1 exome capture array (Roche Nimblegen, Madison, WI, USA), TruSeq Sample Preparation Kit (Illumina, San Diego, CA, USA) and HiSeq2000 Sequencing System (Illumina). Sequence analysis was performed by the Bioinformatics Unit of the Australian Phenomics Facility. A custom analysis pipeline was used to align the sequence reads with the reference genome (C57BL/6 NCBI m37), filter the raw single nucleotide variant (SNV) calls and generate a list of candidate SNVs as described [19]. Deep-sequencing datasets were deposited into the National Center for Biotechnology Information (NCBI) Sequence Read Archive (http://www.ncbi.nlm.nih.gov/sra) with the study accession number SRP020643. The *Chd7* ^Looper^ SNV was PCR-amplified and sequenced using primers TCCTCTGAGATCTAACGAGTCATC and CCAGCAGAGAAGGGAAGAGA under standard conditions and submitted to the AGRF for capillary separation. Sequencing electropherograms were aligned using Seqman v 10.1 software (DNASTAR, Madison, WI, USA).

### Linkage Mapping

BALB/c-Chd7^+/Looper^ mice were crossed to C57BL/6 and the resulting F_1_ offspring were ABR-tested at 8 weeks of age. Those with a click ABR threshold above 40 dB SPL were backcrossed to C57BL/6 to generate 156 F_1_N_1_ offspring. These were ABR-tested at 8 weeks of age and liver DNA isolated as described [20]. Twenty-one affected F_1_N_1_ mice were genotyped for 660 SNPs spaced at 5–10 Mb intervals throughout the genome using the iPLEX Gold method [21], the MassARRAY System (Sequenom, San Diego, CA, USA) and an Autoflex MALDI-TOF mass spectrometer (Bruker, Billerica, MA, USA) at the AGRF. Haplotypes were constructed and regions of heterozygosity identified using Excel v 14.3.4 software (Microsoft, Redmond, WA, USA). F_1_N_1_ mice were genotyped for more finely spaced SNPs on chromosome 4 using the Amplifluor SNPs HT genotyping system FAM-JOE (Merck Millipore, Kilsyth, VIC, Australia) and the primers listed in Table S1. Results were visualized and genotypes assigned using assayauditorEP.xls (Merck Millipore) and excel v 14.3.4 software (Microsoft).

### Histology

For cochlear and middle ear examination, mice were euthanized by intraperitoneal injection of 400 mg/kg ketamine and 80 mg/kg xylazine. After cessation of breathing, PBS was perfused through each animal via a cannula inserted into the left ventricle for 5 min, followed by 10% neutral buffered formalin for 5 min. Cochleae were dissected from the temporal bones and post-fixed for 1 hr at room temperature. Cochleae were washed in tris-buffered saline and decalcified in 10% EDTA for 5 days at 4°C with gentle rolling. Cochleae were oriented in 1% agarose in PBS in 10 mm×10 mm×5 mm cryomolds (Sakura Finetek, Torrance, CA, USA) and paraffin-embedded. 2 µm sections were cut parallel to the modiolus using a microtome and stained with hematoxylin and eosin (H&E). Sections of middle ear cavity were also examined for otitis media.

For eye examination, mice were euthanized via cervical dislocation and eyes fixed in 10% neutral buffered formalin before being embedded in paraffin. 2 µm sagittal sections were cut using a microtome and stained with H&E. Sections were imaged with a DM1000 compound microscope (Leica Microsystems, North Ryde, Australia) and DFC450 C camera (Leica Microsystems).

### X-ray Micro-Computed Tomography (µCT)

µCT was conducted using the Xradia machine MicroXCT-200 (Xradia Inc., Pleasanton, CA, USA), located in the Department of Physics, La Trobe University. An X-ray closed tube source with a Tungsten target was operated at 40 kV tube voltage and power of 10 W. The samples were placed 60–100 mm from the source and 30–50 mm from the detector. The imaging detector was a charge-coupled device camera coupled with a scintillator system and 4x objective lens. The sample was scanned by acquiring 361 projections at equal angles through an angular range of 180 degree using TXMController software (Xradia Inc.). Each projection image was recorded in 7–9 sec. Each image was corrected for the non-uniform illumination in the imaging system, determined by taking a reference image of the beam without sample. A filtered back projection algorithm was then used to reconstruct the acquisition data to create a three-dimensional image using TXMReconstructor software (Xradia Inc.). After the reconstruction process, the distribution of the linear attenuation coefficient was obtained along the section of the sample crossed by the radiation. The total reconstructed volume contained 512×512×512 voxels with the voxel size of 7–11 µm. Three-dimensional (3D) data was viewed with TXM3Dviewer software (Xradia Inc.) and segmented with Avizo-6.2 software (Mercury Computer Systems Inc., Merignac Cedex, France).

### Ocular Examination

Mice were anaesthetised as for ABR testing. To induce dilation of the pupils, 1% Mydriacyl (Alcon Laboratories, NSW, Australia) was applied to the eyes along with an eye lubricant, GenTeal (Novartis, NSW, Australia). A slit lamp was used to examine the anterior segments of each eye. Examination of the retina and optic discs was performed using the slit lamp by placing a cover slip over the cornea. GenTeal between the cornea and cover slip functioned as an ocular medium.

### Behavioural Testing

#### Locomotor cell

The TruScan photo beam activity monitoring system was used to monitor locomotor activity of mice over a 30 min period. Individual mice were placed in locomotor cells (40.6 cm wide×40.6 cm deep×40.6 cm high) in dim (10 lux) lighting. Photo-optic arrays encased the cell containing beams evenly spaced 2.5 cm apart to quantify mouse movements.

#### Swim test

Mice were placed in an inescapable beaker of 25°C water for 1 min. A ceiling-mounted colour CCTV camera (Sony, North Ryde, NSW, Australia) and a DMRES10 DVD recorder (Panasonic, Macquarie Park, NSW, Australia) were used to record behaviour including mobility, sinking and directional rotations.

#### Rotarod

Mice were placed on a rotarod (Ugo Basil, Comerio, VA, Italy) for 4 trials with 1 hr between trials. Trials were conducted under standard lighting of 300 lux. The rotarod speed gradually increased from 4 to 40 rpm during each 5 min trial. The time taken for the mouse to fall off the rotarod was recorded.

#### Digigait

Footprint analysis was conducted using the digigait computerised digital footprint analysis system (Mouse Specifics Inc., MA. USA). Mice were accustomed to a treadmill moving at increasing speeds prior to recording. Four complete step cycles at 15 cm/sec were recorded. Mice were placed in the Perspex chamber on the motorised transparent treadmill and the movement of the paws was captured from the ventral aspect using an A62 high-speed digital video recorder (Basler, Exton, PA, USA) mounted underneath. Gait parameters were calculated using Digigait analysis software.

### Statistical Analysis

Two-way ANOVA, post hoc t-tests, Mann Whitney tests and the Holm Sidak method for multiple testing were performed using Prism 6 software (GraphPad Software Inc.). The Chi-squared test was manually calculated.

## Results

### The *Looper* Phenotype is Caused by a Nonsense Mutation in *Chd7*

**Figure 1:**
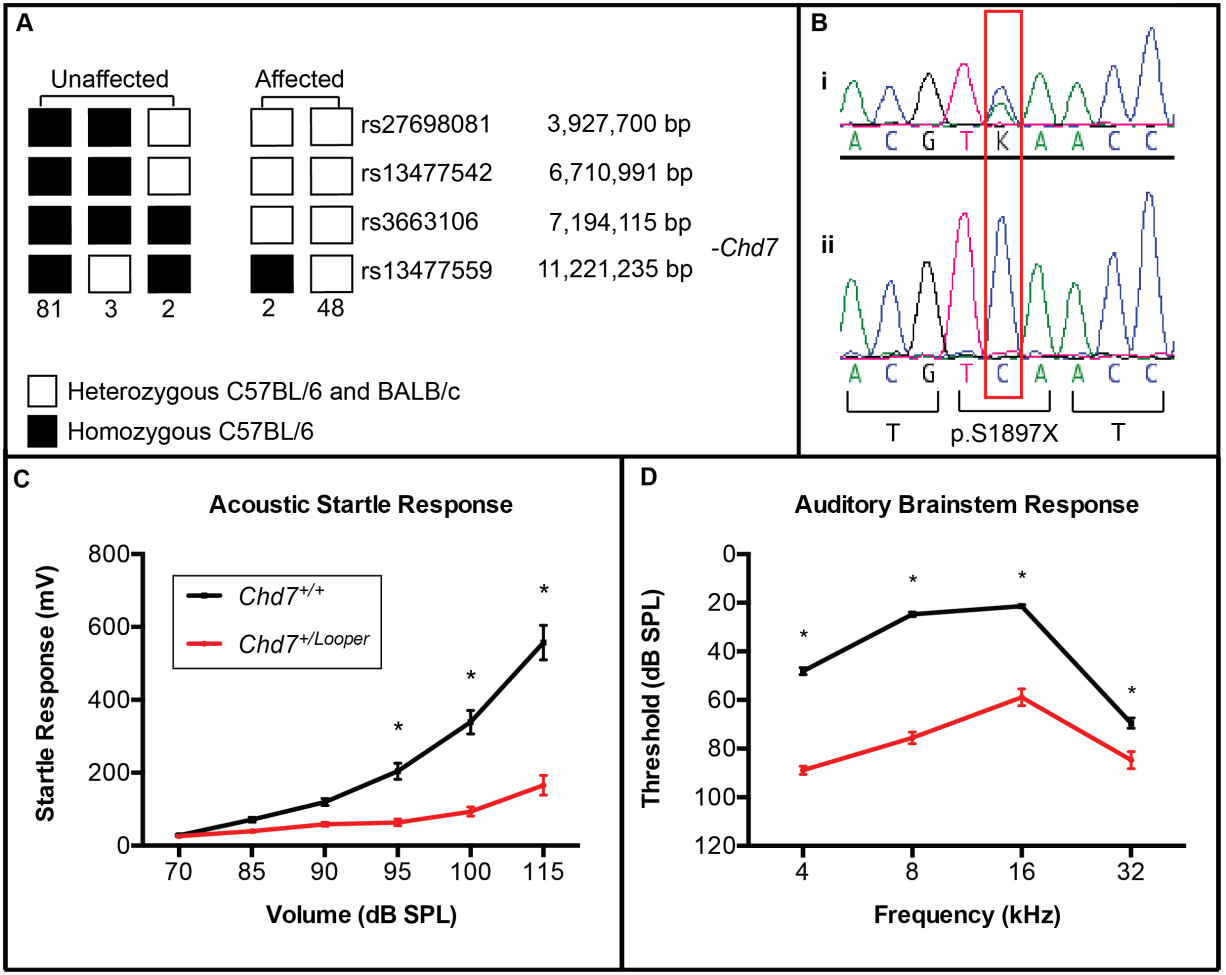
*Looper* mice harbour a nonsense mutation in *Chd7.* **A)** 136 F_1_N_1_ mice were click ABR-tested and classified as unaffected (threshold <35 dB SPL) and affected (threshold >45 dB SPL). Haplotypes for a region of chromosome 4 are illustrated, with the numbers below each haplotype representing the number of mice observed with that haplotype. The region between rs13477542 and rs13477559 was homozygous C57BL/6 in all unaffected mice and heterozygous in all affected mice. This indicated that the *Looper* causative mutation was located between 6,710,991 bp and 11,221,235 bp. **B)** DNA sequence electropherograms of a region of *Chd7* exon 29 in (**i**) an affected *Looper* mouse and (**ii**) an unaffected littermate. The affected mouse was heterozygous for a c.5690C>A mutation, which was predicted to cause premature termination of translation at p.S1897. **C)** Average *Chd7^+/^* ^Looper^ acoustic startle responses (n = 19) were significantly lower than *Chd7^+/+^* controls (n = 20) at 95 to 115 dB SPL. **D)** Average *Chd7^+/^* ^Looper^ ABR thresholds (n = 18) were significantly elevated at all frequencies in comparison to *Chd7^+/+^* controls (n = 21). *p<0.0001 using two-way ANOVA and Fisher’s Least Significant Difference test. Error Bars = SEM.

*Looper* arose in an ENU mutagenesis screen for deaf mice. The G_1_ *Looper* founder mouse failed to startle in response to loud noise and this phenotype proved heritable in a dominant fashion. Meiotic mapping was undertaken to identify the genomic location of the *Looper* mutation. The ABR in response to clicks of 156 F_1_N_1_ mice was assessed, and mice were classified as affected (threshold >45 dB SPL) or unaffected (threshold <35 dB SPL). 20 mice had an intermediate click ABR threshold and were excluded from subsequent analysis. Genotyping of 21 affected F_1_N_1_ mice for 660 SNPs spaced every 5–10 Mb throughout the genome revealed a shared common haplotype between rs13477541 and rs32379175 of proximal chromosome 4 (data not shown). Genotyping of 136 F_1_N_1_ mice for SNPs within this region narrowed the minimal linkage interval to between rs13477542 at 6.7 Mb and rs13477559 at 11.2 Mb (Figure 1A).

Genomic DNA of two BALB/c-*Chd7* ^+/Looper^ N_5_ male mice was subjected to exome enrichment and massively parallel DNA sequencing. 90% of the consensus coding sequence (CCDS) exome was sequenced at least 4-fold and the average depth of sequencing was 84 to 95 fold (Table S2). Between 10 and 14 SNVs were identified in each sample, with 7 SNVs present in both samples, in the following genes: *Ercc5*, *Ankrd44*, *Chd7*, *Mup20*, *Ric3*, *Txlnb* and *Kcnh4*. The only SNV within the minimal linkage interval was the *Chd7* SNV at 8.9 Mb on chromosome 4. Sanger sequencing of DNA from affected and unaffected littermates confirmed this SNV to be a point mutation that segregated with the *Looper* phenotype (Figure 1B). This c.5690C>A change in exon 29 is predicted to result in premature termination of translation of *Chd7* (c.5690C>A, p.S1897X, NCBI reference sequences NC_000070.6, NM_001081417.1, CCDS38689.1, NP_001074886.1).

### *Looper* Mice are Hearing-impaired

*Chd7^+/^* ^Looper^ mice startled less than *Chd7^+/+^* littermates in response to mixed frequency noise (Figure 1C). ABR thresholds of *Chd7^+/^* ^Looper^ mice were significantly elevated between 4 and 32 kHz (Figure 1D). An average threshold shift of 40–50 dB SPL was observed at 4, 8 and 16 kHz, while at 32 kHz, where BALB/c mice are prone to early onset age-related hearing loss [22], an average shift of 15 dB SPL was observed. Interestingly, the average click ABR threshold was significantly higher in *Chd7^+/^* ^Looper^ mice on the BALB/c genetic background than the mixed C57BL/6-BALB/c background (Figure S1).

### *Looper* Mice Display Middle Ear and Vestibular Defects

**Figure 2:**
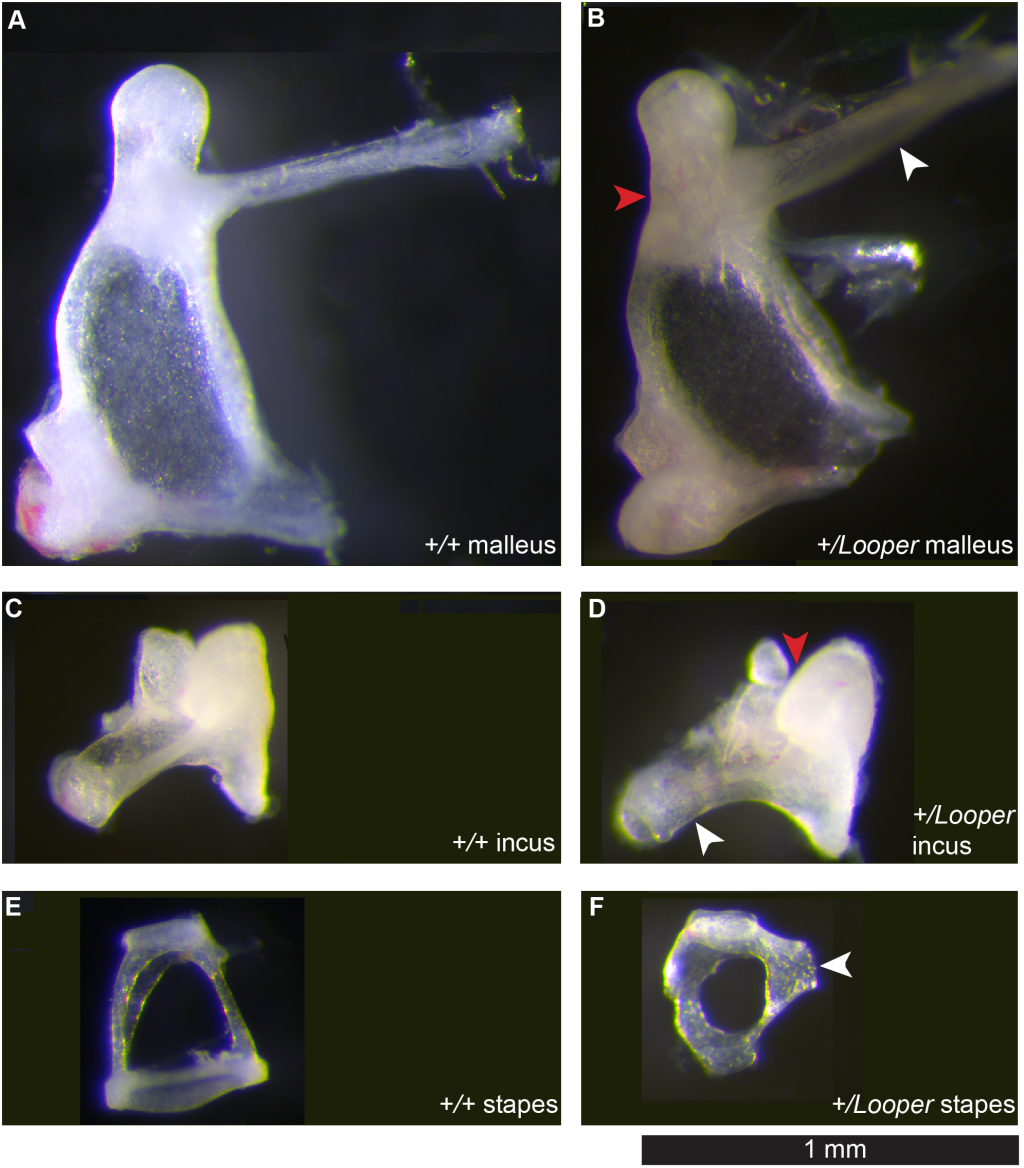
*Looper* ossicles are malformed. Light micrographs of whole mount ossicles. **A)** *Chd7^+/+^* malleus. **B)** *Chd7^+/^* ^Looper^ malleus with slight thickening of the neck (red arrowhead) and a manubrium (white arrowhead) protruding at an acute angle. **C)** *Chd7^+/+^* incus. **D)** *Chd7^+/^* ^Looper^ incus with deformed glenoid cavity (red arrowhead) and malformation of long process (white arrowhead). **E)** *Chd7^+/+^* stapes. **F)** *Chd7^+/^* ^Looper^ stapes, which was small and round with a bone bridge (arrowhead) extending from the tubercle to the otic capsule. Images are representative of 8 *Chd7^+/+^* and 10 *Chd7^+/^* ^Looper^ ears.

**Figure 3:**
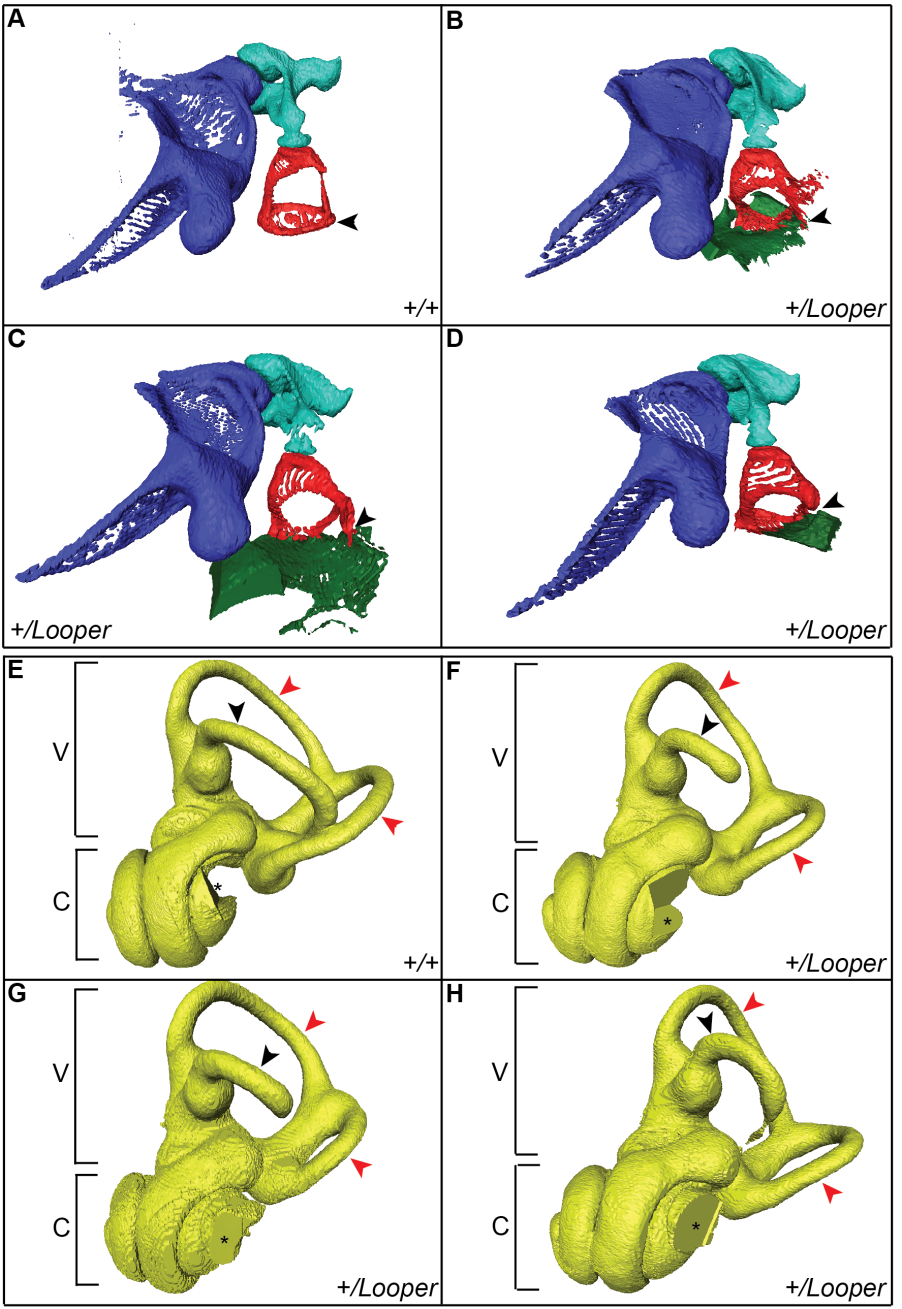
The *Looper* stapes is fused to the oval window and semicircular canals are abnormal. µCT images of left middle (A–D) and inner (E–H) ears of *Chd7^+/+^* and *Chd7^+/^* ^Looper^ mice. **A)** *Chd7^+/+^* and **B–D)** *Chd7^+/^* ^Looper^ malleus (purple), incus (aqua) and stapes (red). The stapes footplate (arrowhead) is fused to the oval window of the cochlea (green) in *Chd7^+/^* ^Looper^ mice. **E)** *Chd7^+/+^* and **F–H)** *Chd7^+/^* ^Looper^ vestibular apparatus (V) and cochlea (C). The lateral semicircular canal (black arrowhead) is incomplete and the posterior and anterior canals (red arrowheads) are hypoplastic in *Cdh7^+/^* ^Looper^ mice. The missing section in each cochlea (*) was an artifact of the imaging/reconstruction process. n = 1 *Chd7^+/+^* and 3 *Chd7^+/^* ^Looper^ mice.

*Chd7^+/^* ^Looper^ ossicles displayed a range of malformations. The *Chd7^+/^* ^Looper^ malleus manubrium protruded at an abnormally acute angle while the neck was thickened and malformed (Figure 2A–B). The *Chd7^+/^* ^Looper^ incus exhibited incomplete development of the long and short process and malformation of the facet (Figure 2C–D). The most striking developmental anomaly was the *Chd7^+/^* ^Looper^ stapes, which was rounded and small with an ectopic bone bridge extending from the tubercle to the otic capsule (Figure 2E–F). µCT imaging revealed fusion of the stapedial footplate to the oval window of the cochlea in all 3 *Chd7^+/^* ^Looper^ samples examined (Figure 3A–D). µCT also revealed incomplete development of the lateral semicircular canal and various degrees of hypoplasia of the posterior and anterior canals (Figure 3E–H). Mid-modiolar sections of the cochlea were examined from four *Chd7^+/+^* and four *Chd7^+/^* ^Looper^ mice and revealed no morphological differences (data not shown).

### *Looper* Mice have Blepharoconjunctivitis but not Coloboma

**Figure 4:**
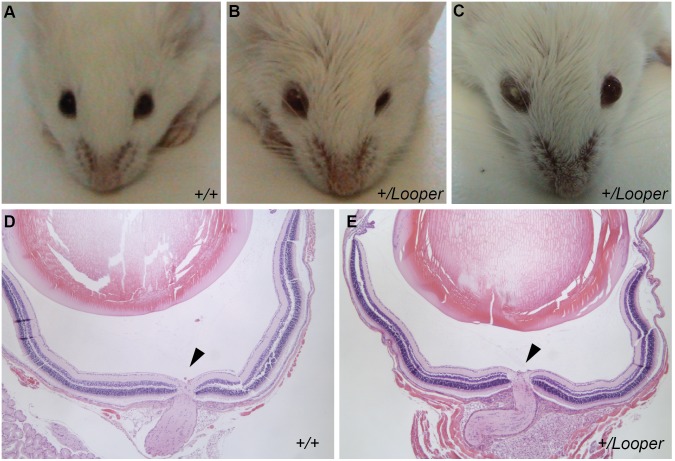
Looper mice display blepharoconjunctivitis. **A–C)** Photograph of the eyes of a *Chd7* ^+/+^ mouse compared with those of two affected *Chd7^+/^* ^Looper^ mice. *Chd7^+/^* ^Looper^ mice exhibited lid oedema, narrowed palpebral fissures (B) and blepharoconjunctivitis (C). Mice ages were 7.5 weeks (A, B) and 10 weeks (C). Images are representative of 11 affected *Chd7^+/^* ^Looper^ mice and 19 unaffected *Chd7^+/+^* mice. **D–E)** Light micrographs of H&E stained sagittal sections of the eye showing the optic nerve head (arrow) for *Chd7^+/+^* (D) and *Chd7^+/^* ^Looper^ (E) mice aged 6 weeks. Mag. 50x. Images representative of n = 4 per genotype.

*Chd7* ^+*/*Looper^ mice exhibited variable, narrowed palpebral fissures and lid oedema, often with one eye severely affected and one eye moderately affected or normal (Figure 4A–C). Eye irritation and probable blepharoconjunctivitis were observed in 11 of 16 *Chd7* ^+*/*Looper^ mice examined. Coloboma was not observed during retinal examination or by histological analysis of the eye (Figure 4D–E).

### *Looper* Mice are Hyperactive and Circle

**Figure 5:**
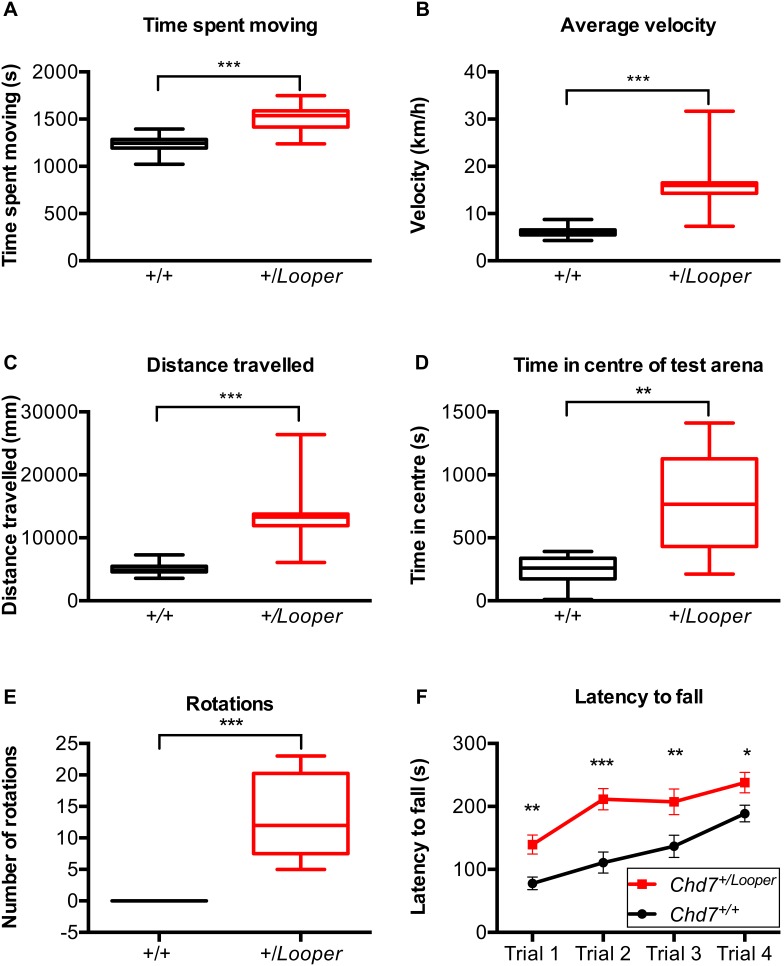
*Looper* mice are hyperactive. **A–E)** Box and whisker plots illustrating the mean and 1–99 percentile range for cohorts of *Chd7^+/^* ^Looper^ and *Chd7^+/+^* littermates for measures taken during Locomotor Cell and Swim testing. **p<0.001, ***p<0.0001 calculated using the Mann Whitney test. **A)** Time spent moving. **B)** Average velocity. **C)** Distance travelled. **D)** Time spent in the centre of the test arena. **E)** Complete rotations made during 1 min of swimming. **F)** Plot of the average latency to fall off the rotor rod over the course of each of four trials, for cohorts of *Chd7^+/^* ^Looper^ and *Chd7^+/+^* littermates. Error Bars  =  SEM. *p<0.05, **p<0.001, ***p<0.0001 calculated using two-way ANOVA and post-hoc Fisher’s Least Significant Difference test (F). n = 12 *Chd7^+/+^* and 12 *Chd7^+/^* ^Looper^ mice.

*Looper* mice exhibited circling behaviour (Video S1). During Locomotor Cell testing, *Chd7^+/^* ^Looper^ mice spent significantly more time moving than *Chd7^+/+^* controls (Figure 5A). On average, *Chd7^+/^* ^Looper^ mice ran twice as quickly (Figure 5B) and travelled twice as far (Figure 5C) as *Chd7^+/+^* controls. This hyperactivity could be the reason for the observed growth delay of *Looper* mice, which were 30% lighter than controls (Figure S2). *Chd7^+/^* ^Looper^ mice were inclined to circle within the centre of the test arena, whilst *Chd7^+/+^* mice preferred the edges of the test arena (Figure 5D). Similar behaviours were observed during the swim test, with *Chd7^+/+^* mice occasionally floating motionless or seeking refuge at the outskirts of the test arena, whilst *Chd7^+/^* ^Looper^ mice swam in circles (Figure 5E, Video S2). *Chd7^+/^* ^Looper^ mice had a longer latency to fall from a rotating rod (Figure 5F) and scored normally in the digigait test (data not shown). These results indicate that directional control, but not motor coordination, is impaired in *Chd7^+/^* ^Looper^ mice.

## Discussion

The *Looper* mutation arose during an ENU mutagenesis screen for deaf mice. Linkage mapping and exome sequencing revealed the *Looper* allele to contain a nonsense mutation at codon 1897 of the *Chromodomain Helicase DNA binding 7* gene (*Chd7*). This nonsense mutation may mediate mRNA degradation (reviewed in [23]). Alternatively, a truncated CHD7 protein may be produced. Such a protein would lack two C-terminal BRK domains (www.ncbi.nlm.nih.gov/protein sequence NP_001264078.1) and may have reduced or dominant negative activity. However, the phenotypic similarity between *Chd7^+/^* ^Looper^ and *Chd7^+/Edy^* mice, which harbour a nonsense mutation at codon 103 [11], indicates that *Chd7* ^Looper^ is likely to be a null allele. The human and mouse amino acid sequences for CHD7 are 95% identical, and although there are no reported mutations that would truncate the human protein at the orthologous residue to *Looper* (p.S1907), multiple pathogenic nonsense mutations have been documented within 10 amino acids [24].

This is the 12^th^ ENU-induced mutation reported for *Chd7*. The large size of the gene (>228,601 bp, 38 exons, reference sequence NC_000070.6) renders it prone to mutation. The same is true for the human orthologue *CHD7,* with 772 reported human mutations in the *CHD7* database (www.chd7.org) [24]. 75% of these mutations are nonsense or frameshift [25]. These pathogenic variants of *CHD7* result in CHARGE syndrome, a complex disease characterised by variable presentation of a range of developmental defects. The pattern of defects observed in human patients is largely reflected in the collection of reported mouse models bearing mutations in *Chd7.* In this manuscript we described in detail the hearing, eye and behavioural abnormalities of the *CHD7*-deficient *Looper* strain. Like other *Chd7* mutant mice, some phenotypes are incompletely penetrant in the *Looper* strain (Table 2). Characterisation of this, and other models of *CHD7*-deficiency will facilitate understanding of the role of this gene and the molecular pathways that it regulates during development.

**Table 2:** Penetrance of *Looper* phenotypes.

Phenotype	Number of *Chd7^+/Looper^* mice examined	*Number of Chd7^+/+^* mice examined
Circling	5/6	0/10
Low acoustic startle response(<200 mV at 115 dB SPL)	15/17	0/22
Hearing impairment(click ABR threshold >40 dB SPL)	18/18	0/22
Stapes malformation (whole mounts)	5/5	0/4
Semicircular canal malformation (microCT)	3/3	0/1
Swimming in circles	12/12	0/12
Hyperactivity (average velocity >10 km/hr)	11/12	0/12
Blepharoconjunctivitis	11/16	0/19

*Chd7^+/L^* ^oo*per*^ mice displayed an attenuated startle in response to white noise, indicating that they were not profoundly deaf. They had an average ABR threshold shift of 50.8 dB SPL at 8 kHz and an average 15.2 dB SPL threshold shift at 32 kHz. This is consistent with conductive hearing loss [26], with higher frequencies still detected due to bone conduction [27]. Cochlear histology was normal but ossicles were abnormal and the stapes fixed. Malformations of the ossicle chain were similar to those reported for other *Chd7* mutants [28]. µCT images of these ossicle abnormalities have not been published before. Unlike the *Chd7^Ome^* mouse [29], otitis media was not observed in *Looper* mice. *Looper* displayed severe hypoplasia of the lateral semicircular canal and variable hypoplasia of the other canals. This is consistent with malformation of the lateral semicircular canal in other *Chd7* mouse mutants [11], [28] and in CHARGE patients, where the lateral canal is always affected and other canals are occasionally mildly affected [30].

*Chd7^+/^* ^Looper^ click ABR thresholds were more severely elevated on the BALB/c genetic background than on the BALB/c.C57BL/6 mixed genetic background. Furthermore, the severity of *Looper* hearing loss and ossicle malformation differed from that of published *Chd7* mutants on different genetic backgrounds [28], [31]. These observations indicate that modifier genes affect the severity of the *Chd7*-deficient hearing phenotype.

An exact cause of the eye inflammation and discharge in *Chd7* mutant mice is yet to be identified. Other *Chd7* mutant mice have been reported to display keratoconjunctivitis sicca or “dry eye” [11]. In contrast, *Looper* mice display blepharoconjuctivitis, which may indicate increased susceptibility to infection or structural changes of the eye socket and lid. Bilateral asymmetry of *Looper* eye phenotypes correlates with CHARGE, but blepharoconjunctivitis does not [32]. Furthermore, ocular coloboma, one of the major diagnostic features of CHARGE was not observed in *Looper* mice.

*Looper* mice spend more time moving at high speed than controls. Hyperactivity has also been noted in CHARGE patients [5]. Vestibular deformity is most likely responsible for the circling behaviour of *Looper* mice. Surprisingly, *Looper* mice demonstrated an enhanced level of motor control during the rotarod test. It is possible the that the innate running behaviour of *Chd7^+/^* ^Looper^ mice may have lead to a higher level of physical conditioning, a confounding factor during this test. However, digigait analysis confirmed that *Looper* motor function was not impaired. Feng *et al*. described reduced neuronal differentiation and abnormal dendritic development of *Chd7*-deficient mice which returned to normal as a result of voluntary running [33]. It is possible that the excessive running of *Chd7^+/^* ^Looper^ mice has a positive effect on their neural pathways.

The excessive activity of *Chd7^+/^* ^Looper^ mice may cause growth delay through reduced food intake and increased energy expenditure. Alternatively, altered hormone levels may be a factor causing growth delay. Postnatal growth delay in CHARGE has been correlated with endocrine anomalies including gonadotrophin and growth hormone deficiencies [34], but the exact cause of growth delay is not understood. Whilst we did not observe heart anomalies or choanal atresia in Looper mice, this may have been due to pre-weaning deaths. 41% (68 of 167) of weaned mice had the *Chd7* ^Looper*/+*^ genotype. This is significantly different from the expected 50% (p<0.05 by Chi-squared test). Heart anomalies, cleft palate and choanal atresia have been identified in embryos of other *Chd7* mutant mice and attributed to early mortality [11].

## Conclusions

*Looper* mice carry an ENU-induced null mutation causing dominant *Chd7* haploinsufficiency. They display phenotypes related to those observed in human CHARGE syndrome including growth retardation, facial asymmetry, hyperactivity, hearing impairment, semicircular canal hypoplasia and fusion of the stapes to the otic capsule and cochlea. Further analysis of the *Looper* strain will facilitate elucidation of the function of *Chd7* and the pathology of CHARGE syndrome.

## Supporting Information

Figure S1 ***Looper*** **hearing loss is modified by genetic background.** Box and whisker plot illustrating the mean and 1–99 percentile range of ABR thresholds in response to clicks for cohorts of affected and unaffected littermates with different genetic backgrounds. Average ABR thresholds were lower for C57BL/6.BALB/c-*Chd7^+/^* ^Looper^ mice (n = 59) than for BALB/c-*Chd7^+/^* ^Looper^ mice (n = 18). ***p<0.0001 calculated using the Mann Whitney test. (C57BL/6.BALB/c-*Chd^+/+^* n = 87, BALB/c-*Chd7^+/+^* n = 21).
(TIFF)

Figure S2 ***Looper*** **mice are growth-delayed. A)** Graphs plotting the average weights of cohorts of male and female *Chd7^+/+^* (n = 14 female and 23 male) and *Chd7^+/^* ^Looper^ (n = 13 female and 12 male) mice each week from 3–12 weeks of age. *p<0.05 calculated using *t –* tests. **B)** Photograph of 53 day old male *Chd7^+/^* ^Looper^ and *Chd7^+/+^* littermates illustrating the difference in size and length. Scale Bar = 5 cm.
(TIF)

Table S1 **Primers used in meiotic mapping.**
(DOCX)

Table S2 **Massively parallel sequencing results.**
(DOCX)

Video S1 ***Looper*** **mice run in circles.** Video of *Chd7^+/+^* (left) and *Chd7^+/^* ^Looper^ (right) littermates, documenting the circling behavior commonly observed for *Chd7^+/^* ^Looper^ mice.
(MP4)

Video S2 ***Looper*** **mice swim in circles.** Video of *Chd7^+/+^* (left) and *Chd7^+/^* ^Looper^ (right) littermates, documenting the swimming in circles commonly observed for *Chd7^+/^* ^Looper^ mice during the 1 min swim test. Representative of n = 12 *Chd7^+/+^* and n = 12 *Chd7^+/^* ^Looper^ mice.
(MP4)
