# Supplementary figures and images for: CHD7 Deficiency in “Looper”, a New Mouse Model of CHARGE Syndrome, Results in Ossicle Malformation, Otosclerosis and Hearing Impairment
Source: PLoS One. 2014 May 19;9(5):e97559. doi: 10.1371/journal.pone.0097559 (PMC4026240; doi:10.1371/journal.pone.0097559)

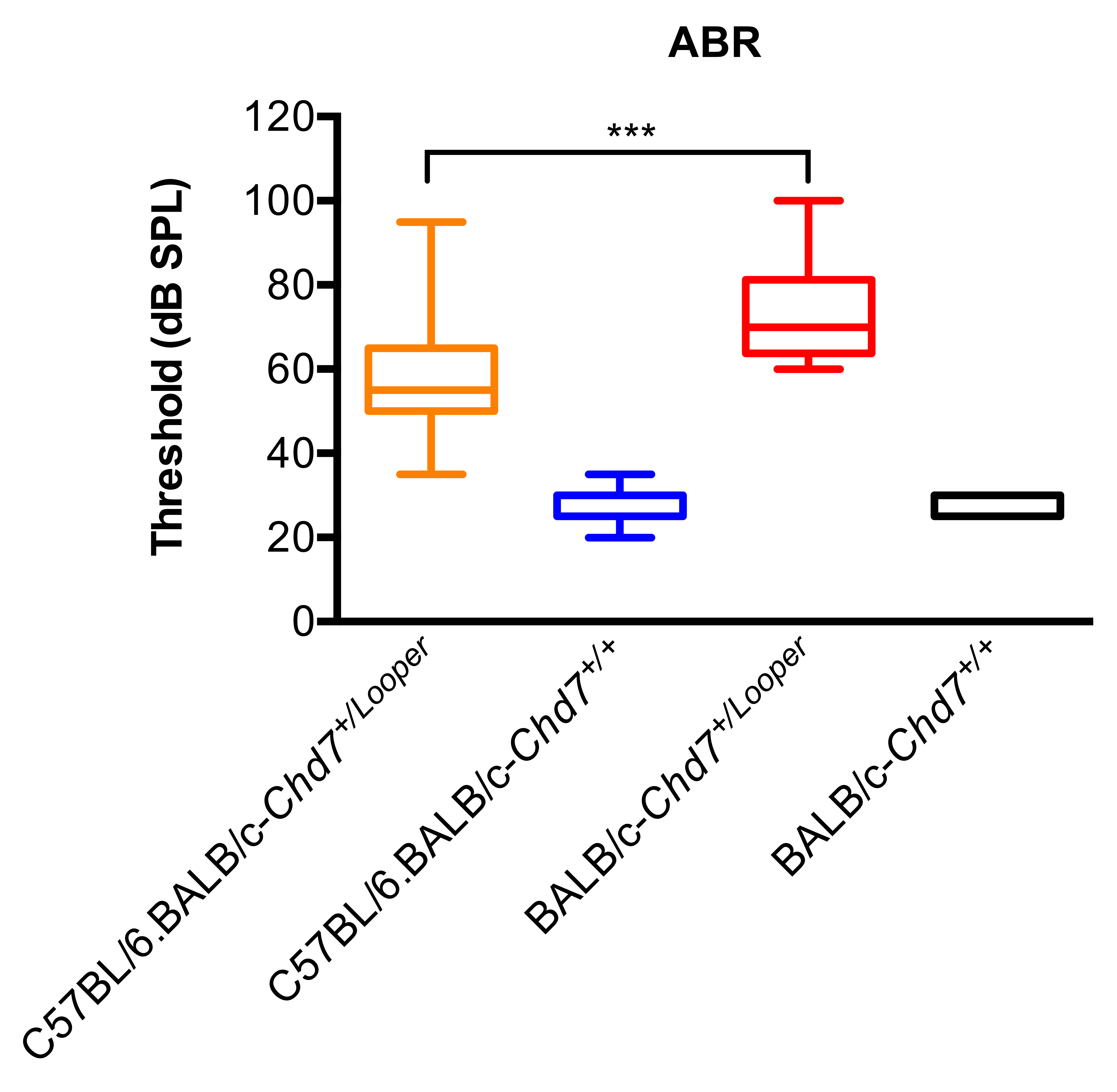

Supplement: Figure S1 — Looper hearing loss is modified by genetic background. Box and whisker plot illustrating the mean and 1–99 percentile range of ABR thresholds in response to clicks for cohorts of affected and unaffected littermates with different genetic backgrounds. Average ABR thresholds were lower for C57BL/6.BALB/c-Chd7+/ Looper mice (n = 59) than for BALB/c-Chd7+/ Looper mice (n = 18). ***p<0.0001 calculated using the Mann Whitney test. (C57BL/6.BALB/c-Chd+/+ n = 87, BALB/c-Chd7+/+ n = 21). (TIFF) [file pone.0097559.s001.tiff]

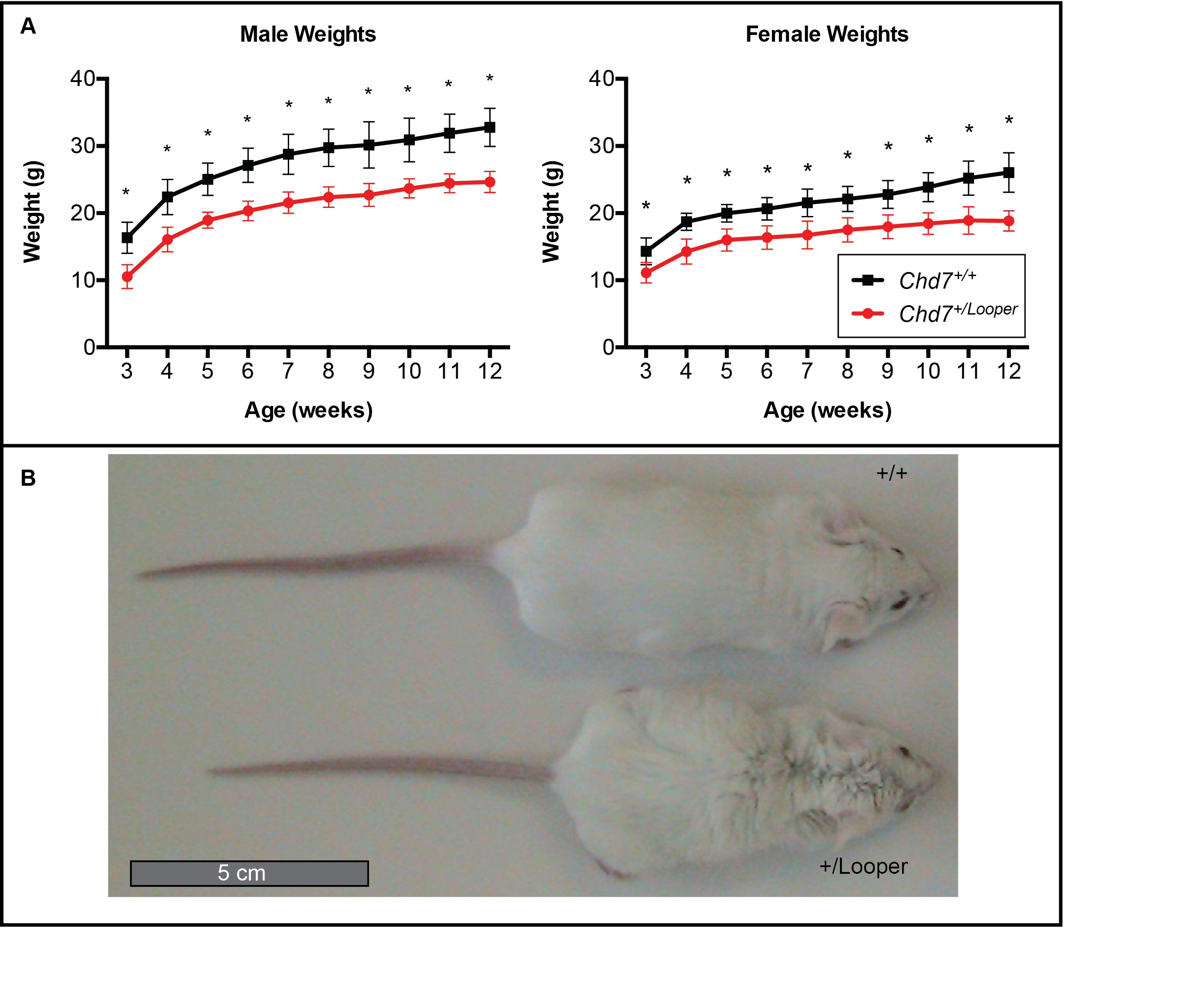

Supplement: Figure S2 — Looper mice are growth-delayed. A) Graphs plotting the average weights of cohorts of male and female Chd7+/+ (n = 14 female and 23 male) and Chd7+/ Looper (n = 13 female and 12 male) mice each week from 3–12 weeks of age. *p<0.05 calculated using t – tests. B) Photograph of 53 day old male Chd7+/ Looper and Chd7+/+ littermates illustrating the difference in size and length. Scale Bar = 5 cm. (TIF) [file pone.0097559.s002.tif]
