# Supplementary material for: CHD7 Deficiency in “Looper”, a New Mouse Model of CHARGE Syndrome, Results in Ossicle Malformation, Otosclerosis and Hearing Impairment
Source: PLoS One. 2014 May 19;9(5):e97559. doi: 10.1371/journal.pone.0097559 (PMC4026240; doi:10.1371/journal.pone.0097559)
Supplement: Table S1 — Primers used in meiotic mapping. (DOCX) [file pone.0097559.s003.docx]

**Table S1: Primers used in meiotic mapping.**

| **Name** | **Sequence** |
| --- | --- |
| rs27698081-F1 | GAAGGTCGGAGTCAACGGATTCTACCACACATGGAGCAAATTCTA |
| rs27698081-F2 | GAAGGTGACCAAGTTCATGCTACCACACATGGAGCAAATTCTG |
| rs27698081-R | CTGACCAGAGCAAGACCTCATA |
| rs13477542-F1 | GAAGGTGACCAAGTTCATGCTTGCATGAAGGGTGGTGTGC |
| rs13477542-F2 | GAAGGTCGGAGTCAACGGATTCTGCATGAAGGGTGGTGTGT |
| rs13477542-R | GAGTTGATTCTGTTTGGTTTAGTGTT |
| rs3663106-F1 | GAAGGTGACCAAGTTCATGCTATTCACTGTAGGGAGACCTTGC |
| rs3663106-F2 | GAAGGTCGGAGTCAACGGATTCATTCACTGTAGGGAGACCTTGT |
| rs3663106-R | CCACCTCAGAACCCAGATACAT |
| rs13477559-F1 | GAAGGTGACCAAGTTCATGCTCAACTTCCCAGAGGTCTTTAGCA |
| rs13477559-F2 | GAAGGTCGGAGTCAACGGATTACTTCCCAGAGGTCTTTAGCG |
| rs13477559 R | CATTCCAGCTTGGACAGCTTT |
