# Supplementary material for: CHD7 Deficiency in “Looper”, a New Mouse Model of CHARGE Syndrome, Results in Ossicle Malformation, Otosclerosis and Hearing Impairment
Source: PLoS One. 2014 May 19;9(5):e97559. doi: 10.1371/journal.pone.0097559 (PMC4026240; doi:10.1371/journal.pone.0097559)
Supplement: Table S2 — Massively parallel sequencing results. (DOCX) [file pone.0097559.s004.docx]

**Table S2: Massively parallel sequencing results.**

| **Mouse** | **Coverage (%)** | **Depth (fold)** | **Number of SNVs** |
| --- | --- | --- | --- |
| Deaf18.D002 | 89.7 | 95 | 10 |
| Deaf18.D012 | 89.7 | 84 | 14 |
